# Molecular dynamics simulation and experimental study of the surface-display of SPA protein via Lpp-OmpA system for screening of IgG

**DOI:** 10.1186/s13568-020-01097-1

**Published:** 2020-09-03

**Authors:** M. Vahed, F. Ramezani, V. Tafakori, V. S. Mirbagheri, A. Najafi, G. Ahmadian

**Affiliations:** 1 Department of Toxico/Pharmacology, School of Pharmacy, Shahid Beheshti University of Medical Sciences, Niayesh Highway, Valiasr Ave, Tehran, 6153-14155 Iran; 2 Pharmaceutical Sciences Research Center, Shahid Beheshti University of Medical Sciences, Niayesh Highway, Valiasr Ave, Tehran, 14155-1817 Iran; 3 Physiology Research Center, Faculty of Medicine, Iran University of Medical Sciences, Tehran, Iran; 4 Department of Cell & Molecular Sciences, Faculty of Biological Sciences, Kharazmi University, Tehran, Iran; 5 PhD Student in Fisheries Products Processing Group, Faculty of Fisheries and Environmental Sciences, Gorgan University of Agricultural Sciences and Natural Resources, Gorgan, Iran; 6 Department of Environmental and Industrial Biotechnology, National Institute of Genetic Engineering and Biotechnology (NIGEB), P.O.BOX: 14965/161, Tehran, 1497716316 Iran; 7 Present Address: Department of Cell and Molecular Biology, Uppsala University, P.O. Box 256, 751 05 Uppsala, Sweden

**Keywords:** Protein A–IgG interaction, Surface-displayed OmpA, Protein interaction, Molecular dynamics

## Abstract

Staphylococcal protein A (SpA) is a major virulence factor of *Staphylococcus aureus*. *S. aureus* is able to escape detection by the immune system by the surface display of protein A. The SpA protein is broadly used to purify immunoglobulin G (IgG) antibodies. This study investigates the fusion ability of Lpp′-OmpA (46–159) to anchor and display five replicate domains of protein A with 295 residues length (SpA295) of *S. aureus* on the surface of *Escherichia coli* to develop a novel bioadsorbent. First, the binding between Lpp’-OmpA-SPA295 and IgGFc and the three-dimensional structure was investigated using molecular dynamics simulation. Then high IgG recovery from human serum by the surface-displayed system of Lpp′-OmpA-SPA295 performed experimentally. In silico analysis was demonstrated the binding potential of SPA295 to IgG after expression on LPP-OmpA surface. Surface-engineered *E. coli* displaying SpA protein and IgG-binding assay with SDS-PAGE analysis exhibited high potential of the expressed complex on the *E. coli* surface for IgG capture from human serum which is applicable to conventional immune precipitation.

## Introduction

*Staphylococcus aureus* protein A (SpA) is a surface component of the bacteria (Ton-That et al. 1999) which play a role as a key virulence factor for the *S. aureus* pathogenesis. This is achieved through strong binding of the SpA to the Fcγ domain of various species of IgG (Sjodahl 1977) and the Fab domain of VH3-type of B cell receptors (Cary et al. 1999). SpA is comprised of five Ig-binding domains arranged as (IgBDs) E, D, A, B, and C (Boyle 1990). Protein A is able to bind, with high affinity, to most IgG subclasses of human, cows, pigs, hamsters, horses, pigs, and rabbits and with low affinity to chicken, goat, rat IgG subclasses (Hadji-Ghasemi et al. 2003). This protein has been widely used for quantitative and qualitative immunological techniques including different kinds of ELISA (Lofdahl et al. 1983; Tashiro and Montelione. 1995).

Display of heterologous proteins on the bacterial surface has been demonstrated as a multi-strategy approach to develop an efficient vaccine for *S. aureus* development (Kim et al. 2010; Kalyanasundram et al. 2015), screening of antibody libraries (Cavallari 2017), development of whole-cell bioadsorbents (Tafakori et al. 2012), and biosensors (Furst et al. 2017). Chimeric protein system of the Lpp′-OmpA is used as an anchor and loads heterologous proteins onto the Gram-negative bacterial surface (Yang et al. 2008a, b; Georgiou et al. 1996). Lpp′-OmpA consists of the first nine aminoacids of the *E. coli* lipoprotein (Lpp) which is fused to the residues 46–159 of the OmpA porin protein family to anchor bacterial cell wall envelope (Francisco et al. 1992; Tafakori et al. 2014).

We examined the possibility of surface displaying of SpA295 via a Lpp′-OmpA system and its binding capability to IgG_FC_ using bioinformatics and computational tools, which was confirmed by the experimental methods. SpA protein was successfully immobilized on the *E. coli* surface using an Lpp’-OmpA (46–159) fusion system to develop an efficient method for purification and immunoprecipitation of IgG antibodies.

## Materials and methods

### Computer modeling

The structure of SpA protein according to the amino acid sequence in this study that comprises five repeat domains of 295 amino acid residues in length (SpA_295_) was predicted by ModWeb server (Pieper et al. 2014). The nucleotide sequence of the Lpp′-ompA-Spa construct was submitted to genebank with the accession number: MT680197. The Geometric coordinates of X-ray crystallography of IgG were obtained from RCSB protein data bank with the access code: 4ZNC.

### Computational condition of docking and molecular dynamic simulation

To provide the stable structure of Lpp′-OmpA-SPA295, this complex was subjected to molecular dynamic (MD) simulation for 30 ns. MD simulation was performed by GROMACS 5.0.5 software (Van Der Spoe et al. 2005) and OPLSAA force field similar to that shown in the previous study (Ghahremanifard et al. 2018; Hashemzadeh et al. 2018; Fasehee et al. 2018) The molecules were placed in a dodecahedron box containing the water molecule in TIP3P model. In order to create the ionic conditions of 0.15 molar, water molecules were replaced with Na^+^ and Cl^−^ ions and the total charge of system was neutralized. The initial energy minimization was performed using the steepest descent algorithm. After that, the NVT simulation was performed for 50 ps and followed by the NPT ensemble for 30 ns. In order to maintain the temperature of 300° K and the pressure of 1 bar, nose-hover thermostat and Berendsen barostat were used, respectively. R = 1.2 was considered for electrostatic and van der Waals interactions.

The stable structure of Lpp′-OmpA-SPA295 from primary MD simulation was used to investifate the interaction of this structure with IgG. For this purpose, the HDOCK server (Yan et al. 2017) was employed to investigate Lpp’-OmpA-SpA295-IgG interaction according to default parameters of protein–protein free docking hybrid algorithm of template-based modeling.

The complex obtained from the HDOCK server was subjected to 30 ns molecular dynamic simulation under the conditions used for the primary MD simulation.

All structures visualized by the Discovery studio. The number of hydrogen bond (H-bond) formed between acceptor and donor atoms is measured using the geometrical criteria of a donor–acceptor distance less than 3.5 Å by RING 2.0 web server (The RING 2.0 web server for high quality residue interaction networks).

### Materials used in experimental model

List of the primer pairs, bacterial strains and plasmids used in this study are listedin Table 1. The SpA gene were amplified from the genomic DNA of *S. aureus* (ATCC 6538) as a template, the Pfu DNA polymerase (Fermentas, Germany) and primers shown in Table 1. For design of growth curves and optimization tests, bacterial cultures were grown in Luria–Bertani (LB) medium containing 50 mg/ml kanamycin sulfate. Isopropyl ß-d-thiogalactopyranoside (IPTG) was used to induce the expression of recombinant protein n. In this study Human serum was used for binding analysis.

**Table 1:** Primers, plasmids and strains used in this study

Primer, plasmid or strains	Description or genotype	Source or reference
Primer		
PAF—*Eco*RI	GGGG G AAT TC T GCA AAT GCTGCGCAACAC	MWG
PAR—*Xho*I	GGGG CTCGAG TTTTGGTGCTTGAGCATCGT	MWG
P1 (LPO1-F) *Nde*I	GGGGCATATGAAAGCTACTAAACTGGTACTGGGCAACCCGTATGTTGGCTTTGAAATGGG	Tafakori et al. (2012)
LPOTA, *Eco*RI	GGGGGAATTCGCTCCCGGAATGCCGTTGTCCGGACGAGTG CC	Tafakori et al. (2012)
PET 26b PET 26b-lOAE (pLOAa)	T7 promoter, an N-terminal pelB signal sequence for potential periplasmic localization, plus optional C-terminal His·Tag Vector for construction and expressing of chimeric protein containing lpp′-ompA, Elongatus and Chitin Binding domain	Qiagen Novagene Constructed in this study Constructed in this study
Strain		
BL21 DE3	F-ompT gal dcm lon hsdSB (rB- mB-) λ (DE3 [lacI lacUV5-T7 gene 1 ind1 sam7 nin5]	Stratagene
Top 10 *Staphylococcus aureus*	F′[lacIq Tn10 (tetR)] mcrA Δ (mrr-hsdRMS-mcrBC) _ϕ_ 80lacZΔM15 ΔlacX74 deoR nupG recA1 araD139 ∆(araleu) 7697 galU galK rpsL(StrR) endA1 λ−	Invitrogen

### Construction of plasmids, and protein expression

The Lpp′-OmpA fragment was amplified by PCR using LPOA1 and LPOTA primers to construct pLOAa plasmid, previously made from the pET-LOA plasmid, containing a combining of the first nine N-terminal amino acids of Lpp and amino acids 46 to 159 of OmpA were used as a template. The 381 bp PCR product was digested with *Nde*I–*Eco*RI restriction enzymes, followed by ligation into the previously digested pET26b vector.

PCR was carried out as per following conditions: Initial denature for 5 min at 94C, amplify 30 cycles of 45 s at 94 °C, annealing foe 45 s at 68 °C, extension for 60 s at 72 °C, and final extension for 10 min at 72 °C on an MWG AG, Biotech, Primus 96 system (Germany). For gene cloning, the truncated SpA gene was amplified using primers PAF, PAR from ~ 890 bp, S. *aureus* genome as a template (100 ng/25 µl), and purified. To prepare the final construct of plasmid, SpA fragment (890 bp) and pLOAa were digested by *Eco*RI and *Xho*I and followed by ligation into the plasmid pLOAa. The vector was labeled as pLOA-PA pLOA-PA. Transformation of the vector into *E. coli* was carried out. It used CaCl_2_-mediated procedure. Overnight cultures of recombinant bacterial inoculated into 1 l of fresh LB medium which contains 50 μg/ml kanamycin sulfate. To express fusion proteins, cultures were induced by using IPTG 0.1 mM, for 16 h. Centrifugation was used at 10000*g* for 2 min to harvest the cells.

### Preparation of surface-engineered *E. coli* displaying SpA protein

A single colony of the recombinant *E. coli* harboring plasmids pLOA-PA was grown overnight in the LB media containing 35 μg/ml of kanamycin and was then inoculated into the fresh medium and continued for 8 h further incubation.

The bacterial culture was collected by centrifugation at 5000×*g* for 10 min followed by a washing step with 10 ml PBS buffer by resuspended in PBS following a washing step. This was repeated for three more times.

The bacteria pellet was resuspended in 10 ml of PBS + 0.02% sodium azide, transferred to a 250 ml erlenmeyer flask and stirred at room temperature. Formaldehyde solution was added to give 1.5% final concentration and stirring was continued for 80 min at room temperature. Formaldehyde was removed by washing the suspension with 15 ml of 1X PBS. After discarding the supernatant, the pellet was washed in PBS + 0.02% sodium azide as a 10% (w/v) followed by a 5 min centrifugation at 5000×*g*. After resuspension of the bacterial pellet in a 100 mM Tris–HCl buffer pH 8 containing 10% glycerol, it was stored at 4 °C.

### IgG-binding assay

The affinity and IgG-binding ability of the protein A-displaying *E. coli* was examined using IgG-binding assay with rabbit sera. The surface-engineered *E. coli* was washed with 1 mL of suspension buffer (100 mM Tris–HCl pH 8). The pH of rabbit sera was increased to 7.5–8 by 1 M Tris of pH 8 and it then added to the bacterial suspension followed by incubation for 1 h at 4 °C. The surface-engineered bacteria were washed with 100 mM Tris–HCl of pH 8. Elution buffer (100 mM glycine of pH 3 containing 1 M KCl) was used to release the bound IgGs from surface of the bacterial. Eluted fraction was dialyzed in 10 mM Tris–HCl and then resolved on SDS-PAGE prepared according to Laemmli (1970) and then stained with Coomassie Brilliant Blue R-250. *E. coli* transformed with the parental plasmid pLpp′-OmpA without IgG-binding domain of SpA was used as a negative control.

## Results

### Complex binding of SpA with IgG_Fc_

**Fig. 1:**
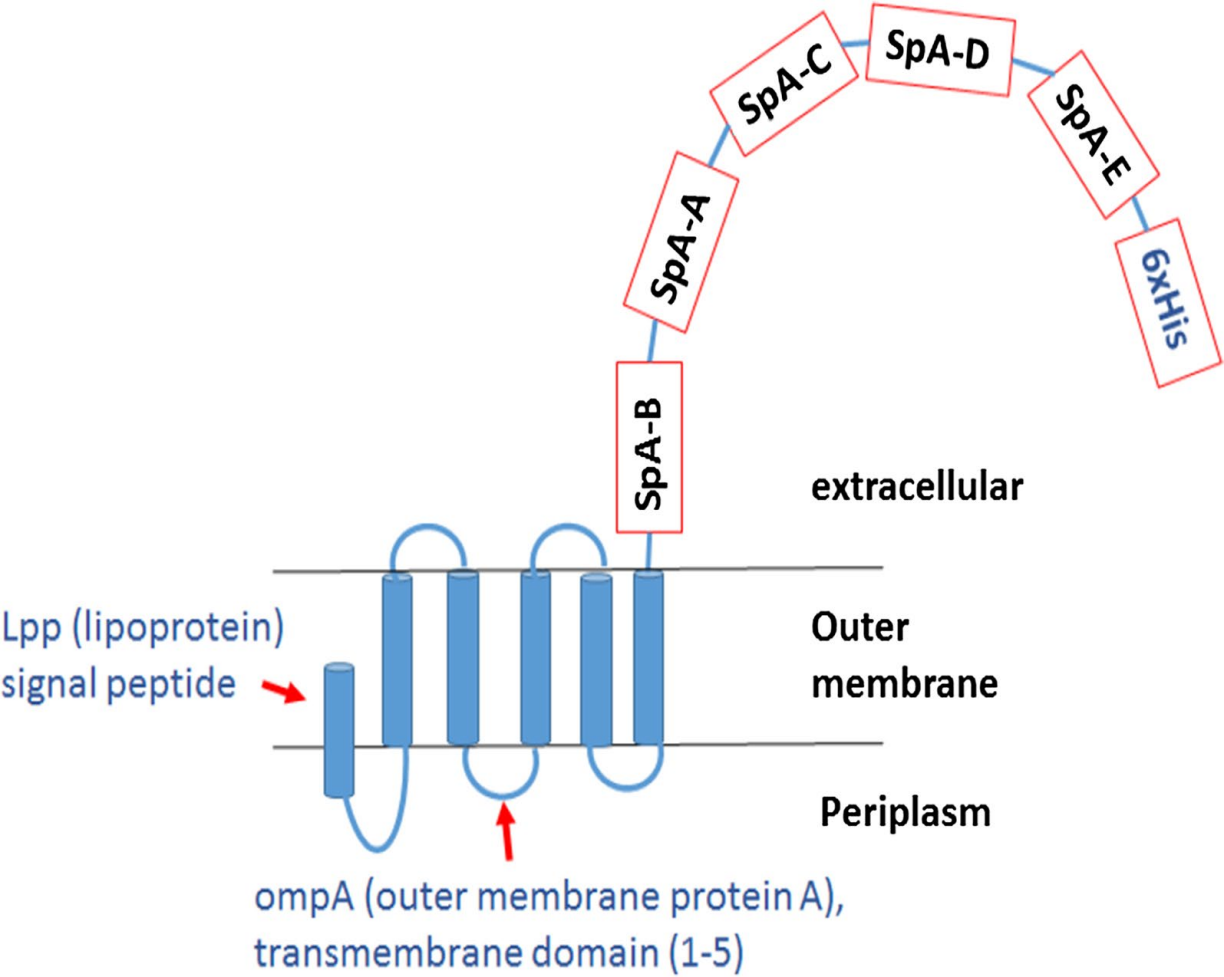
Schematic illustration of Lpp′-OmpA, SpA_295_ complex

**Fig. 2:**
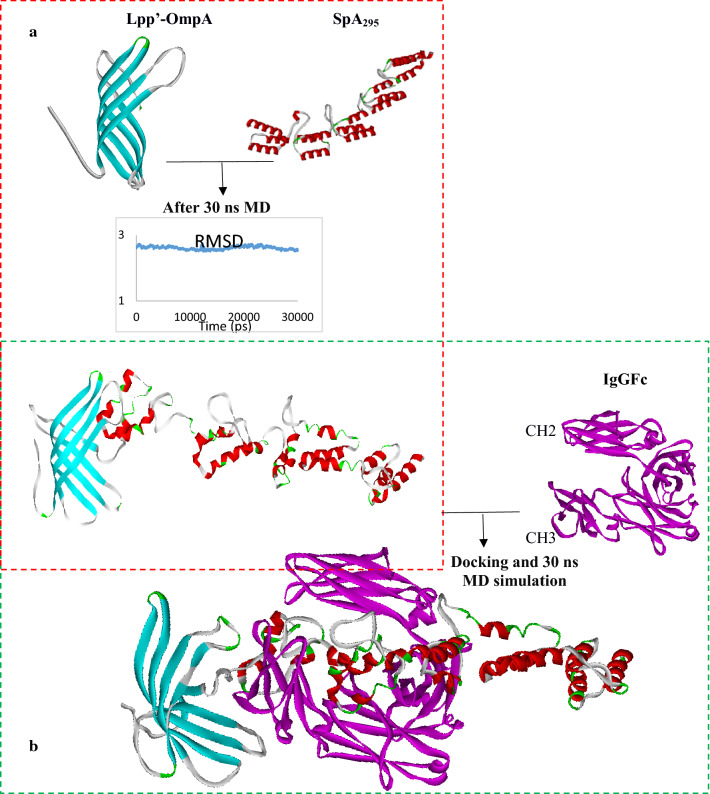
**a** The primary structure of Lpp’-OmpA, SpA_295_ and its complex after 30 ns molecular dynamics, RMSD plot of protein structure compared to the primary structure. **b** Lpp′-OmpA-Spa_295 -_and IgG_FC_ and their cpmlex after docking and 30 ns molecular dynamics simulation

We evaluated the formation of the pLpp′-OmpA-SpA_295_-IgG_FC_ structure (Fig. 1) using a high-throughput computational approach. The root mean square deviation (RMSD) and the predicted structure of Lpp’-OmpA-SpA_295_ are presented in Fig. 2a. The RMSD shows the stability of the system after 30 ns of MD simulation. Binding site of the correct structure obtained from molecular dynamic to IgG_FC_ determined by docking. The docking cluster scoring models are according to the energy range from the minimum energy interaction of protein–protein complexes. We selected the complex with the lowest free energy from docking to for following molecular dynamic simulation for 30 ns that the resulted structure is in Fig. 2b.

**Fig. 3:**
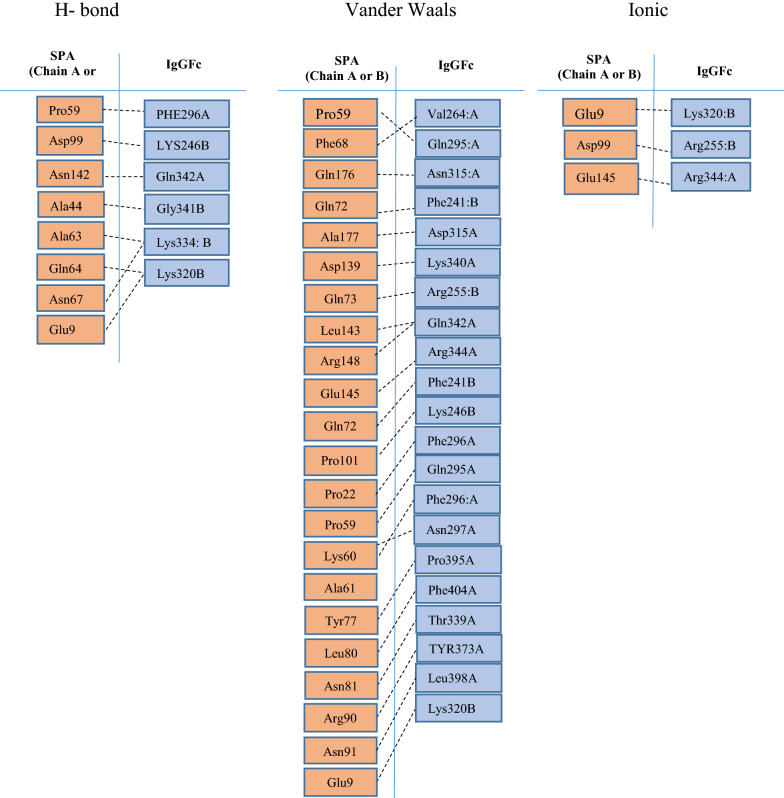
Illustration of H-bonds, Vander Waals and ionic interaction of SpA_295_ with IgG_Fc_ observed after 30 ns molecular dynamic simulation (yellow broken lines represent H-bonds between two residues)

The H-bonds among SpA_295_ with IgG_Fc_ were displayed in Fig. 3. Pro59, Asp99, Asn142, Ala44, Ala63, Gln64, Asn67, Glu9 in SPA involved in H- bond with IgG. van der Waals and ionic bonding between IgGFc and SpA295 are also seen in the Fig. 3. Glu9, Asp99, Glu145 make ionic bond with IgG.

**Fig. 4:**
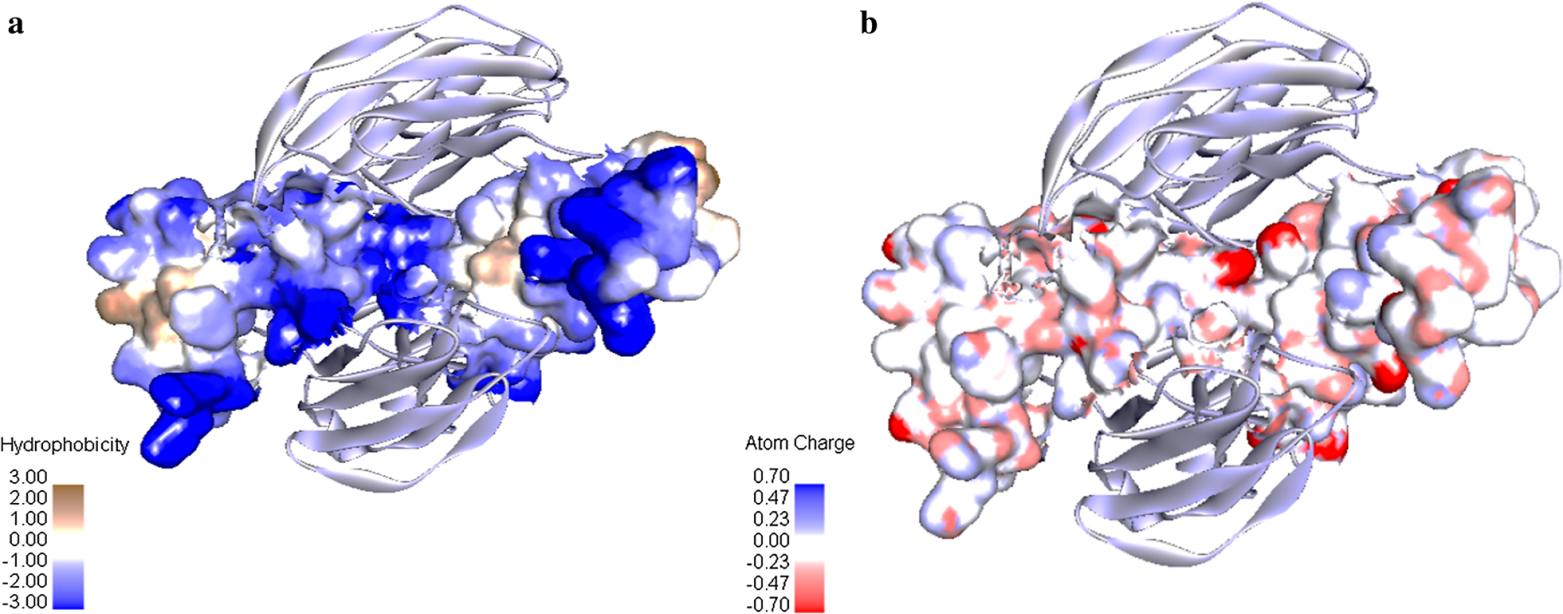
**a** The hydrophobic surface display of interaction of SpA with IgG_Fc_, **b** The electrostatic surface display of interactions SpA with IgG_Fc_

Hydrophobic sites on the surface of SpA in the region that interact with IgG (Fig. 4a) and the surface electrostatic potential of SpA are observed in Fig. 4b. As can be seen, the number of hydrophilic amino acids and also, negatively charged amino acids are more common in the interaction site.

### Plasmid construction and protein expression

Surface attachment of the protein A (SpA) containing E, D, A, B and C domains (Additional file 1: Figure S1) on the surface of *E.* *coli* BL21 (DE3) was successfully done using the Lpp′-OmpA system. The construct of Lpp′-ompA-Spa in pET26b plasmid was made. DNA sequence of pLpp′-ompA-Spa construct cloned in pET26b, and protein sequence of p Lpp′-ompA-Spa construct are provided in Additional file 1: Figure S2.

Construction of the pET26b plasmid was verified by restriction enzyme digestions (Additional file 1: Figures S3–S5), and DNA sequencing. Amplification of protein A performed using PCR reactions at different temperatures (Additional file 1: Figure S6). Detection of non-recombinant and recombinant plasmids pET26 was performed by enzymatic digestion (Additional file 1: Figure S7) and electrophoretic mobility shift assay (Additional file 1: Figure S8).

**Fig. 5:**
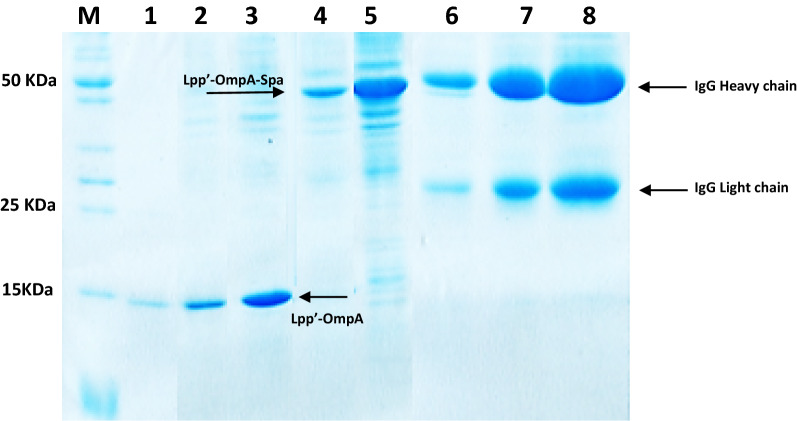
SDS-PAGE analysis showing efficiency of *E. coli* Lpp′-ompA-SpA compared to the Protein A-agarose, stained by Coomassie Brilliant Blue R. M:Protein marker, 1–3: different concentrations of expressed recombinant Lpp′-ompA was loaded, 4–5: different concentrations of expressed recombinant Lpp’-ompA-Spa was loaded, 6–7: different concentrations of the purified rabbit IgG from serum using recombinant *E. coli* Lpp′-ompA-SpA after elution was loaded, 8: purified rabbit IgG from serum using protein A-agarose as a control

The expression of the fusion truncated SpA and the control protein was carried out using *E. coli* BL21 DE3 and IPTG as an inducer. The protein expression by *E. coli* transformed with recombinant plasmids pET-LOA (Fig. 5, line 1–3) and pLOA-PA (Fig. 5, line 4–5) was evaluated using SDS-PAGE analysis (Additional file 1: Figures S9 and S10).

The recombinant truncated Protein A contains five Ig-binding regions of protein A and a 6× His-tag at the C-terminus. To prevent nonspecific binding to IgG, the albumin binding region and other regions present in SpA was removed to ensure specific IgG binding. Immobilized metal affinity chromatography (IMAC) was used to purify the recombinant C-terminus 6X his-tag fusion of the SpA. Meanwhile, using anti-His-tag antibody the fusion protein A can be detected. The recombinant Protein A is ideal for immunoprecipitation and purification of antibodies as it is able to binds to most human and mouse IgG subclasses of human and mouse.

### Binding assay for SpA-displayed recombinant *E. coli*

The recombinant SpA-displaying *E. coli* adsorbent that developed in this work was used for purification of the IgG from rabbit serum. After binding and two steps of washing to remove nonspecific proteins, the protein A eluted from the bioabsorbent contained mainly IgG molecules.

The presence of the protein bands corresponding to the IgG heavy and light chains (about 50 kDa and > 25 kDa, respectively) on polyacrylamide gel further verifies that IgG has adhered to the protein A immobilized on the recombinant *E. coli* surface (Fig. 5 line 6 and 7). The results of the IgG purification were also comparable with that of the one achieved by commercial protein A- agarose in which the SpA is immobilized on the surface of this polymer. Eluted IgG from protein A-agarose support prepared in this study was qualitatively comparable to purified IgG using immobilized protein A agarose matrix supports by SDS-PAGE analysis as shown in Fig. 5 line 8. It is notable that protein A bind and extracts the intact form of IgG from serum by attaching to its Fc region, without changing the conformation and structure of IgG as it occurs when a reducing agent such as DTT which breaks S–S bond is present in the buffers and cause dissociation of heavy chain and light chain of IgG.

## Discussion

Antibody purification using Protein A (SpA), immobilized to different solid surfaces, is commonly used in research laboratories or commercial antibody manufacturing. Increasing the stability of SpA protein under harsh treatment/washing conditions is an important factor to increase the yield and quality of purified antibodies (Rigi et al. 2019; Cherf and Cochran 2015). One of the major strategies to increase proteins stability is display of proteins on the surface of a live cell as an alternative to classic protein immobilization approach (Lozančić et al. 2019; Grewal, et al. 2016).

The classical immobilization of recombinant proteins on the surface of a matrix (Khodaei et al. 2018) is sometimes challenging because the protein may lose its conformation and consequently its function. However, anchoring proteins is a mild approach to immobilize heterologous proteins to outer membranes of the cell. In this way, the host cell produces the heterologous protein while covalently attaching it to its surface. In this worke, we used the *E. coli* surface display method to express SPA protein for IgG isolation. The immunoadsorbent generated from *E. coli* surface display, in addition to the other benefits mentioned, can be quickly generated in a cost-effective way and stored lyophilized at room temperature, which will be stable for several months and reduces the cost of downstream processes in industry as well.

The efficiency of surface display systems and the correct and efficient protein folding and its stability is highly related to the specifications of the carrier protein, passenger protein, and fusion method (Yang. et al. 2008a, b; Barrett et al. 2019). LPP-ompA is an efficient surface display system that has been developed and applied for various applications (Fasehee et al. 2018; Rigi et al. 2014; Tafakori et al. 2012). In this research project using Lpp′-OmpA as an SPA anchor, in the initial design we performed physico-chemical and structural studies on chimeric protein using molecular dynamics tools to ensure the strength and stability of this new structure on the cell surface.

Computational analysis showed that the surface expression of SpA_295_ creates a stable structure and does not form undesirable bonds with the Lpp′-OmpA surface protein, and maintains its binding structure to IgG_Fc_. Furthermore, the analysis displayed in the binding of Lpp′-OmpA-SpA297 complex to IgG_F_c in which aminoacids involve in Vander Waals interaction, hydrogen binding and ionic binding.

In the experimental work, surface expression of this new the recombinant protein system by five replicate domains of protein A on the surface of *Escherichia coli* BL21 and the power of IgG separation confirmed computer simulation findings. The absorption rate under the five SpA repeatitive domain systems was extremely high. Higher IgG-binding yield is demonstrated by SDS-PAGE analysis which can be used as a suitable alternative to conventional immune precipitates.

The surface display system used in this study seems to be suitable for surface engineering of *E. coli* to immobilize various ligands and proteins in their active state. An advantage of the current system over conventional commercially available immobilization matrix is simplicity, high production rate, easier production and handling processes, and the lower cost of preparing the.

The matrix created in this work is able to separate IgG from human sera as a functional assay and considering the yield, purity of IgG and the cost of producing the matrix, this system can be used to develop an efficient immunoadsorbent.

We have shown that the displayed protein domains with specific functions of IgG purification at the cellular surface are accessible in binding studies. Furthermore, with the whole cell as a matrix, the proteins have proven to be more stable, therefore making downstream processes of associated preparations and protein purification redundant. Further investigation on this system is required to achieve higher production rates and specificity at the industrial scale.

## Supplementary information

**Additional file 1: Figure S1.** Schematic diagram Of Protein A domains. **Figure S2.** A) DNA Sequence of p Lpp’-ompA-Spa construct cloned in pET26b, and B) protein sequence of p Lpp’-ompA-Spa construct. **Figure S3.** Schematic diagram showing the strategy for making recombinant plasmid pLpp’-ompA-Spa. **Figure S4.** Investigation of electrophoretic mobility shift assay of different recombinant clones on 1% agarose gel to confirm cloning using quick check extraction method. **Figure S5.** Investigation of enzymatic cleavage of recombinant pLpp’-ompA plasmids with *Nde*I and *Eco*RI restriction enzymes by 1% agarose gel electrophoresis. **Figure S6.** Amplification of protein A using PCR reactions at different temperatures from 54 to 57 from left to right. **Figure S7.** Enzymatic digestion of non-recombinant and recombinant plasmids pET26. **Figure S8.** Investigation of electrophoretic mobility shift assay of different recombinant clones on 1% agarose gel to confirm cloning using quick check extraction method. **Figure S9.** Relative expression and purification of Lpp’-ompA and Lpp’-ompA-Spa in *E. coli* (BL21-DE3) under control of the T7 promoter. **Figure S10.** Expression of Lpp’-ompA-Spa construct in *E. coli* (BL21-DE3).

## Data Availability

Not applicable.

## Abbreviations

SpA: Staphylococcus aureus protein A
IgG: Immunoglobulin G
ompA: Outer membrane protein A
Lpp'-ompA: Truncated outer membrane protein A
IPTG: Isopropylthiogalactopyranoside
IgBDs: Ig-binding domains
LB medium: Luria–Bertani medium
