# Supplementary material for: Molecular dynamics simulation and experimental study of the surface-display of SPA protein via Lpp-OmpA system for screening of IgG
Source: AMB Express. 2020 Sep 3;10:161. doi: 10.1186/s13568-020-01097-1 (PMC7471224; doi:10.1186/s13568-020-01097-1)
Supplement: Supplementary file 1 — Additional file 1: Figure S1. Schematic diagram Of Protein A domains. Figure S2. A) DNA Sequence of p Lpp’-ompA-Spa construct cloned in pET26b, and B) protein sequence of p Lpp’-ompA-Spa construct. Figure S3. Schematic diagram showing the strategy for making recombinant plasmid pLpp’-ompA-Spa. Figure S4. Investigation of electrophoretic mobility shift assay of different recombinant clones on 1% agarose gel to confirm cloning using quick check extraction method. Figure S5. Investigation of enzymatic cleavage of recombinant pLpp’-ompA plasmids with NdeI and EcoRI restriction enzymes by 1% agarose gel electrophoresis. Figure S6. Amplification of protein A using PCR reactions at different temperatures from 54 to 57 from left to right. Figure S7. Enzymatic digestion of non-recombinant and recombinant plasmids pET26. Figure S8. Investigation of electrophoretic mobility shift assay of different recombinant clones on 1% agarose gel to confirm cloning using quick check extraction method. Figure S9. Relative expression and purification of Lpp’-ompA and Lpp’-ompA-Spa in E. coli (BL21-DE3) under control of the T7 promoter. Figure S10. Expression of Lpp’-ompA-Spa construct in E. coli (BL21-DE3). [file 13568_2020_1097_MOESM1_ESM.docx]

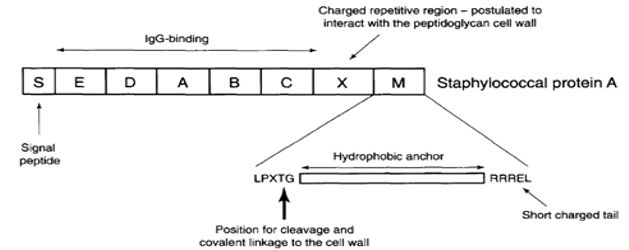


**Domains present in the full-length spa**

**Figure S1. Schematic diagram Of Protein A domains.** But in this study truncated spa protein containing five IgG-binding domain (amino acid 1-327) Was used.

1. **DNA sequence >ompA-SpA**

*Nde*I- Lpp'-ompA-linker- *Eco*RI-spa-*Xho*I-6X histag-tga

atg aaa gct act aaa ctg gta ctg ggc

aacccgtatgttggctttgaaatgggttacgactggttaggtcgtatgccgtacaaaggcagcgttgaaaacggtgcatacaaagctcagggcgttcaactgaccgctaaactgggttacccaatcactgacgacctggacatctacactcgtctgggtggcatggtatggcgtgcagacactaaatccaacgtttatggtaaaaaccacgacaccggcgtttctccggtcttcgctggcggtgttgagtacgcgatcactcctgaaatcgctacccgtctggaataccagtggaccaacaacatcggtgacgcacacaccatcggcactcgtccggacaac

GGC ATT CCG GGA GCG AAT TCA

GCAAA TGCTGCGCAA CACGATGAAG CTCAACAAAA TGCTTTTTATCAAGTCTTAA ATATGCCTAA CTTAAATGCT GATCAACGCA ATGGTTTTAT CCAAAGCCTTAAAGATGATC CAAGCCAAAG TGCTAACGTT TTAGGTGAAG CTCAAAAACT TAATGACTCTCAAGCTCCAA AAGCTGATGC GCAACAAAAT AACTTCAACA AAGATCAACA AAGCGCCTTCTATGAAATTT TGAACATGCC TAACTTAAAC GAAGCGCAAC GCAATGGTTT CATTCAAAGTCTTAAAGACG ATCCAAGCCA AAGCACTAAC GTTTTAGGTG AAGCTAAAAA ATTAAACGAATCTCAAGCAC CGAAAGCTGA CAACAATTTC AACAAAGAAC AACAAAATGC TTTCTATGAAATCTTGAACA TGCCTAACTT GAACGAAGAA CAACGCAATG GTTTCATCCA AAGCTTAAAAGATGACCCAA GTCAAAGTGC TAACCTATTG TCAGAAGCTA AAAAGTTAAA TGAATCTCAAGCACCGAAAG CGGATAACAA ATTCAACAAA GAACAACAAA ATGCTTTCTA TGAAATCTTACATTTACCTA ACTTAAACGA AGAACAACGC AATGGTTTCA TCCAAAGCTT AAAAGATGACCCAAGCCAAA GCGCTAACCT TTTAGCAGAA GCTAAAAAGC TAAATGATGC ACAAGCACCAAAAGCTGACA ACAAATTCAA CAAAGAACAA CAAAATGCTT TCTATGAAAT TTTACATTTACCTAACTTAA CTGAAGAACA ACGTAACGGC TTCATCCAAA GCCTTAAAGA CGATCCTTCAGTGAGCAAAG AAATTTTAGC AGAAGCTAAA AAGCTAAACG ATGCTCAAGC ACCAAAActcgag CACCACCACCACCACCACTGA

**B) Protein sequence**

MKATKLVLGNPYVGFEMGYDWLGRMPYKGSVENGAYKAQGVQLTAKLGYPITDDLDIYTRLGGMVWRADTKSNVYGKNHDTGVSPVFAGGVEYAITPEIATRLEYQWTNNIGDAHTIGTRPDN

GIPGANSANAAQHDEAQQNAFYQVLNMPNLNADQRNGFIQSLKDDPSQSANVLGEAQKLNDSQAPKADAQQNNFNKDQQSAFYEILNMPNLNEAQRNGFIQSLKDDPSQSTNVLGEAKKLNESQAPKADNNFNKEQQNAFYEILNMPNLNEEQRNGFIQSLKDDPSQSANLLSEAKKLNESQAPKADNKFNKEQQNAFYEILHLPNLNEEQRNGFIQSLKDDPSQSANLLAEAKKLNDAQAPKADNKFNKEQQNAFYEILHLPNLTEEQRNGFIQSLKDDPSVSKEILAEAKKLNDAQALPETG

Figure S2. A) DNA Sequence of p Lpp'-ompA-Spa construct cloned in pET26b, and B) protein sequence of p Lpp'-ompA-Spa construct.


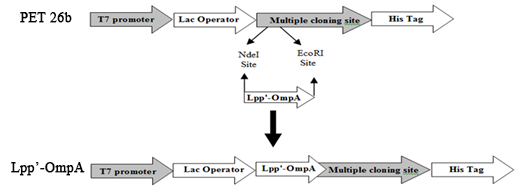


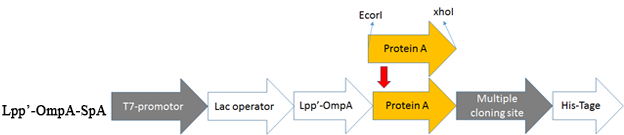


Figure S3- **Schematic diagram showing the strategy for making recombinant plasmid pLpp'-ompA-Spa.** In this study of the following primers were used.

LPO1, (with *Nde*I site) Forward: 5'GGGGCATATGAAAGCTACTAAACTGGTACTGGGCAACCCGTATGTTGGCTTTGAAATGGG 3'

LPOTA, (with *Eco*RI site) Reverse: 5'GGGGGAATTCGCTCCCGGAATGCCGTTGTCCGGACGAGTGCC-3'

Primers to clone PA (32-327) after Lpp’-ompA in pET26b

PAF *Eco*RI 29 mer Tm= 58

GGGG G AAT TC T GCA AAT GCTGCGCAACAC

PAR *Xho*I no stop codon- FUSED TO 6X His Tm= 58

5 GGGG CTCGAG TTTTGGTGCTTGAGCATCGT

**Figure S4- Investigation of electrophoretic mobility shift assay of different recombinant clones on 1% agarose gel to confirm cloning using quick check extraction method**. Column 1, 3, 4 : Negative control (plasmid pET26b). Columns 2, 5, 6, 7, 8 and 9 of recombinant pLpp'-ompA plasmids. The first row is genomic DNA and the second row are plasmids.

1 2 3 4 5 6 7 8 9

Genomics DNA

Plasmid DNA


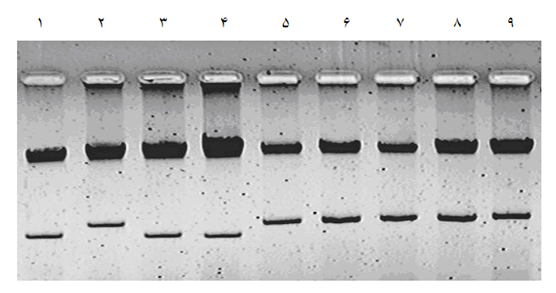

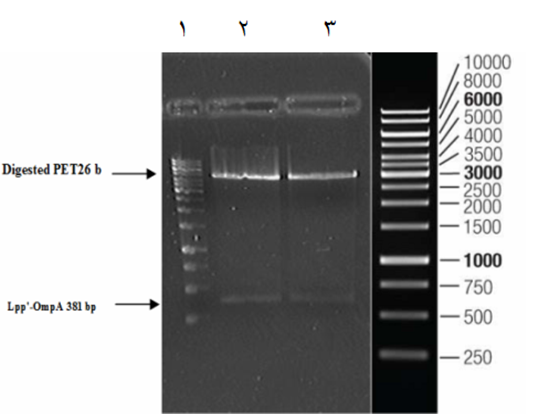


1 2 3 3

**Figure S5- Investigation of enzymatic cleavage of recombinant pLpp'-ompA plasmids with *Nde*I and *Eco*RI restriction enzymes by 1% agarose gel electrophoresis**. 1: 1 kb marker. 2 and 3: pLpp'-ompA plasmid digested with NdeI and EcoRI. A fragment of Lpp'-ompA is released.


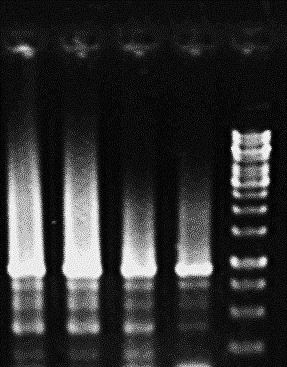


**Figure S6- Amplification of protein A using PCR reactions at different temperatures from 54 to 57 from left to right.**

1 2 3 4 5 M

**Figure S7 - Enzymatic digestion of non-recombinant and recombinant plasmids pET26.**1- vector pET26b undigested
2- just vector pET26b digestion with *Xho*I
3- vector pET26b Enzymatic digestion with *Xho*I / *Nde*I
4- Enzymatic digestion of recombinant Lpp'-ompA-Spa with *Xho*I
5- Enzymatic digestion of recombinant Lpp'-ompA-Spa with *Xho*I / *Nde*I. A fragment of Lpp'-ompA-Spa is released.


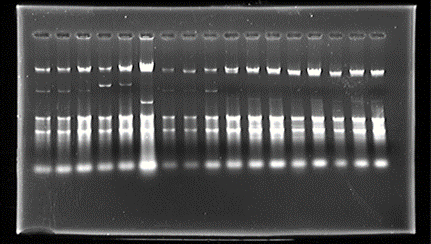


1 2 3 4 5 6 7 8 9

**Figure S8 - Investigation of electrophoretic mobility shift assay of different recombinant clones on 1% agarose gel to confirm cloning using quick check extraction method**. Column 6: Negative control (non-recombinant plasmid pET26b). Columns 4, 5 of the recombinant plasmids (Lpp'-ompA-Spa). Columns of other numbers of recombinant plasmids (Lpp'-ompA). The first row from the top of the gel is related to genomic DNA and the second row is plasmids extracted using quick check extraction.


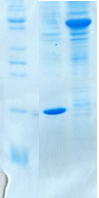


1 2 3

50 kD

15 kD

**Figure S9 - Relative expression and purification of Lpp'-ompA and Lpp'-ompA-Spa in *E. coli* (BL21-DE3) under control of the T7 promoter.** 1- Ladder protein, 2- Lpp'-ompA construct, 3- Lpp'-ompA-Spa construct (six hours after

**Figure S10 - Expression of Lpp'-ompA-Spa construct in *E. coli* (BL21-DE3).** the sizes of the Molecular Weight Marker in Kilodalton is shown in the left-hand side.
